# Selective loss of Cx40 and conduction abnormalities underlie enhanced atrial fibrillation susceptibility in a HFpEF model in male mice

**DOI:** 10.14814/phy2.70952

**Published:** 2026-06-04

**Authors:** Kohei Kawajiri, Satoshi Iwamiya, Leo Yaga, Masahiro Yamazoe, Kensuke Ihara, Yurie Soejima, Tetsuo Sasano

**Affiliations:** ^1^ Department of Cardiovascular Medicine Institute of Science Tokyo Tokyo Japan; ^2^ Department of Pathology and Anatomical Sciences Institute of Science Tokyo Tokyo Japan

**Keywords:** atrial fibrillation, connexin 40, electrophysiological remodeling, heart failure with preserved ejection fraction, optical mapping

## Abstract

Heart failure with preserved ejection fraction (HFpEF) is frequently complicated by atrial fibrillation (AF), yet the mechanisms underlying AF vulnerability prior to or independent of extensive structural remodeling remain incompletely understood. In this study, we investigated atrial electrophysiological remodeling in a murine HFpEF model induced by combined high‐fat diet and L‐NAME administration. The model reproduced key HFpEF features, including obesity, hypertension, preserved systolic function, diastolic dysfunction, and impaired exercise capacity. Transesophageal atrial pacing revealed a marked increase in AF inducibility in HFpEF mice. Ex vivo optical mapping demonstrated prolonged action potential duration, global conduction slowing, and increased conduction heterogeneity in the left atrium. Notably, atrial fibrosis, inflammation, and atrial enlargement were absent. Instead, both gene and protein expression analyses showed a selective downregulation of connexin 40 (Cx40), while connexin 43 expression was preserved. Immunohistochemistry confirmed reduced expression and altered localization of Cx40 in atrial myocardium. These findings indicate that Cx40 downregulation and gap junction dysfunction precede structural remodeling and constitute a primary electrophysiological AF substrate. Our results highlight impaired atrial conduction as a primary mechanism of AF vulnerability in this metabolic HFpEF model and provide new insights into the pathogenesis of HFpEF‐associated AF that may inform future preventive strategies.

## 1 INTRODUCTION

Heart failure with preserved ejection fraction (HFpEF) represents a major global health burden with rising prevalence (Dunlay et al., 2017). Notably, left atrial (LA) dysfunction is more strongly associated with mortality in HFpEF than in heart failure with reduced ejection fraction (HFrEF) (Melenovsky et al., 2015). LA remodeling in HFpEF has traditionally been regarded as a secondary phenomenon resulting from increased left ventricular filling pressure or chronic systemic inflammation, particularly in patients with obesity and metabolic comorbidities (Campbell et al., 2024; Redfield & Borlaug, 2023).

Atrial fibrillation (AF) is a common and severe comorbidity in HFpEF, and their coexistence significantly worsens clinical outcomes (Zakeri et al., 2013). In patients with HFpEF, LA compliance and contractile function progressively decline with increasing AF burden, creating a vicious cycle that facilitates AF initiation and progression (Reddy et al., 2020). Indeed, AF progression correlates closely with the extent of LA fibrosis in HFpEF patients (Lee et al., 2024). While advanced HFpEF with chronic AF typically exhibits extensive atrial fibrosis, accumulating evidence suggests that AF can occur in patients with minimal fibrosis (Marrouche et al., 2014), raising the possibility that electrophysiological alterations may precede or contribute to AF vulnerability independently of structural remodeling.

Despite these clinical insights, the mechanisms of AF vulnerability prior to or independent of significant structural remodeling remain incompletely understood. Experimental evidence from other cardiac disease models suggests that electrophysiological alterations, including ion channel remodeling and gap junction dysfunction, may develop before or independently of structural changes (Filgueiras‐Rama et al., 2012; Morillo et al., 1995; Zakeri et al., 2013), but whether similar mechanisms operate in HFpEF remains unclear. However, investigating the role of these electrical changes in traditional heart failure animal models is challenging as many models involve rapid, severe pressure overload, induces to early and extensive fibrosis that obscures the potential contribution of electrical remodeling to AF susceptibility (Withaar et al., 2021; Zhang et al., 2021).

To directly investigate these electrical alterations in the absence of extensive fibrosis, we employed a metabolic HFpEF model induced by high‐fat diet (HFD) combined with Nω‐nitro‐L‐arginine methyl ester (L‐NAME), a nitric oxide synthase inhibitor (Schiattarella et al., 2019). This combination induces systemic endothelial dysfunction and hypertension, and cardiac metabolic disturbances, recapitulating the phenotype characteristic of HFpEF, including ventricular diastolic dysfunction. Although the ventricular electrophysiological properties of this model have been partially characterized (Mesquita et al., 2025), its atrial electrophysiological properties remain largely unexplored. Importantly, this model reproduces key features of HFpEF with only mild ventricular fibrosis, providing an ideal platform to evaluate the atrial electrical substrate before extensive structural remodeling occurs (Mesquita et al., 2025). In this study, we applied a high‐resolution optical mapping system to Langendorff‐perfused murine hearts, enabling precise evaluation of atrial conduction and repolarization properties. The primary objective of this study was to comprehensively characterize atrial electrophysiological properties, AF susceptibility, and the underlying molecular mechanisms in this murine metabolic HFpEF model.

## 2 METHODS

### 2.1 Experimental animals

All animal procedures were conducted in accordance with the Guidelines for the Care and Use of Laboratory Animals published by the National Research Council (The National Academies Press, 8th edition, 2011). All experimental protocols were approved by the Institutional Animal Care and Use Committee of the Institute of Science Tokyo under protocol numbers #A2021‐014 and #A2024‐103. Male C57BL/6JJcl mice (9–10 weeks old) were purchased from CLEA Japan, Inc. The HFpEF phenotype was induced by feeding mice a high‐fat diet 32 (HFD; 60% kilocalories from fat, CLEA Japan) and providing L‐NAME (0.5 g·L^−1^; Nacalai Tesque, Kyoto, Japan, 24540‐74) in the drinking water for the indicated experimental period (Schiattarella et al., 2019). Control mice were fed a standard chow diet (Rodent Diet, CLEA JAPAN, CE‐2) and provided normal drinking water. The pH of the L‐NAME–containing water was adjusted to 7.4 before administration. Mice were maintained on HFD and L‐NAME treatment for a total of 15 weeks to establish the HFpEF model. Mice were euthanized by cervical dislocation for all terminal procedures, including tissue collection for RNA extraction and optical mapping experiments.

### 2.2 In vivo physiological measurements

Tail‐cuff blood pressure recordings and transthoracic echocardiography were performed to assess in vivo cardiovascular function. Blood pressure in awake mice was measured using a tail‐cuff plethysmograph (BP‐98AL; Softron, Tokyo, Japan) according to the manufacturer's instructions. Ultrasound echocardiography was conducted using a VINNO 6LAB system (VINNO Technology, Suzhou, China) in a blinded manner. Mice were anesthetized with <1.0% isoflurane, and body temperature was continuously monitored and maintained at 37.0°C using a heating pad and a heat lamp during the procedure.

### 2.3 Exercise exhaustion test

After 3 days of acclimatization to treadmill exercise, an exhaustion test was performed as previously described with modifications (Schiattarella et al., 2019). Mice ran uphill at a 5° incline on a motorized treadmill (LE8706MTS; Panlab, Barcelona, Spain), beginning with a warm‐up phase consisting of 5 m·min^−1^ for 2 min, 8 m·min^−1^ for 2 min, and 10 m·min^−1^ for 2 min. Following the warm‐up, the exercise test was initiated by increasing the treadmill speed by 2 m·min^−1^ every 2 min until the mouse reached exhaustion. Exhaustion was defined as either (1) failure to resume running within 10 s after direct contact with the electric stimulus grid, or (2) three or more stops within 1 min.

### 2.4 In vivo electrophysiological study

Electrodes were attached to the limbs, and single‐lead ECG recordings were obtained using a Powerlab system (ADInstruments, Dunedin, New Zealand). Signals were digitized and analyzed with LabChart software (ADInstruments). Programmed stimulation for AF induction was performed in vivo using a custom‐made bipolar pacing catheter inserted into the murine esophagus under 1.5% isoflurane anesthesia. Body temperature was continuously monitored and maintained at 37.0°C. The pacing catheter was positioned in the esophagus dorsal to the left atrium, where the lowest capture threshold was obtained. All pacing stimuli were delivered at twice the capture threshold. Atrial stimulation was performed using a programmed stimulation protocol modified from previously published methods (Sasano et al., 2006; Wakimoto et al., 2001). Briefly, up to three extrastimuli were delivered following 10 drive beats at a pacing cycle length of 100 ms. If no arrhythmia was induced by extrastimuli, burst pacing was applied by delivering 50 beats at 10–50 Hz. AF was defined as a sustained tachyarrhythmia lasting >1 s, characterized by irregular RR intervals and an altered A‐wave morphology compared with sinus rhythm.

### 2.5 Optical mapping

Optical mapping was performed in a blinded manner with minor modifications from previously published protocols (Ihara et al., 2018). Mice were intraperitoneally injected with unfractionated heparin (200 IU) and euthanized by cervical dislocation. The excised hearts were immediately connected to a Langendorff perfusion system and stained with 5.0 μM di‐4‐ANEPPS (Santa Cruz Biotechnology, Dallas, TX, USA), followed by administration of 250 μM blebbistatin (Toronto Research Chemicals, Toronto, ON, Canada) to suppress motion artifacts. Fluorescence signals were recorded using a complementary metal‐oxide semiconductor camera (MiCAM ULTIMA; BrainVision, Tokyo, Japan) at a sampling rate of 0.2 ms and a spatial resolution of 66 μm. Ex vivo electrophysiological pacing was performed by placing a custom‐made pacing electrode on the right atrium. Pacing was delivered at a voltage twice the capture threshold, using constant pacing cycle lengths of 60, 80, 100, and 120 ms. Action potential duration at 90% repolarization (APD₉₀) was calculated for each pixel. Conduction velocity (CV) was derived from isochronal activation maps as described previously (Morley et al., 1999). Action potential (AP) rise time was defined as the interval from the onset of depolarization—determined as the point of maximal second derivative—to the peak AP deflection. Electrophysiological heterogeneity was quantified using the coefficient of variation, calculated as the standard deviation divided by the mean value within a defined region of interest (ROI). For all analyses, the ROI was consistently positioned within the left atrium, excluding boundary regions. Group comparisons were performed using mean values within the ROI for each map.

### 2.6 RNA extraction and quantitative PCR

Total RNA was extracted from heart tissue using the RNeasy Mini Kit (QIAGEN, Hilden, Germany, 74106) according to the manufacturer's instructions. Complementary DNA (cDNA) was synthesized using the High‐Capacity cDNA Reverse Transcription Kit (Thermo Fisher Scientific, Waltham, MA, USA, 4374966). Quantitative PCR (qPCR) was performed on a StepOnePlus real‐time PCR system (Thermo Fisher Scientific) using THUNDERBIRD SYBR qPCR Mix (Toyobo, Osaka, Japan, QPS‐201). Gene expression levels were normalized to *Gapdh* using the ΔΔCt method. The primer sequences used in this study are listed in Table S1.

### 2.7 Western blotting for atrial protein expression

Protein extracts from heart tissue were prepared using radioimmunoprecipitation assay buffer (Nacalai Tesque). Protein concentrations were determined with the BCA Protein Assay Kit (Thermo Fisher Scientific). Equal amounts of protein (10 μg per lane) were separated by SDS–polyacrylamide gel electrophoresis using TGX FastCast gels Acrylamide Kit (Bio‐Rad Laboratories, Hercules, CA, USA, 161‐0175) and transferred onto polyvinylidene fluoride membranes (Immobilon—P^SQ^, MilliporeSigma, Burlington, MA, USA, ISEQ00010) with the Trans‐Blot Turbo transfer system (Bio‐Rad). Membranes were blocked with 5% skim milk in Tris‐buffered saline with Tween 20 (Nacalai Tesque) and incubated overnight at 4°C with the following primary antibodies: goat anti‐Cx40 (Santa Cruz Biotechnology, sc‐20466; 1:1000), rabbit anti‐Cx43 (Proteintech, Rosemont, IL, USA, 26980‐1‐AP; 1:1000), mouse anti‐GAPDH (Santa Cruz Biotechnology, sc‐32233; 1:10,000). After washing, membranes were incubated with horseradish peroxidase–conjugated secondary antibodies: anti‐mouse IgG (Cell Signaling Technology, Danvers, MA, USA, 7076S; 1:10,000), anti‐rabbit IgG (Cell Signaling Technology, 7074S; 1:10,000), anti‐goat IgG (Dako, Santa Clara, CA, USA, P0449; 1:10,000). Chemiluminescent signals were visualized using Chemil‐Lumi One L (Nacalai Tesque, 07880‐70) and detected using the iBright CL1500 imaging system (Thermo Fisher Scientific). Band intensities were quantified using ImageJ software (National institute of Health, Bethesda, MD, USA) and normalized to GAPDH.

### 2.8 Histological assessment

The excised hearts were immersed in 10% buffered formalin and the paraffin sections were subjected to Masson's trichrome staining. The stained sections were photographed using BZ‐X710 microscope and BZ‐X Analyzer software version 1.4.1.1 (Keyence, Osaka, Japan), and the fibrotic areas were quantified by using the ImageJ software (National institute of Health). The proportion of fibrotic area was calculated as the ratio of the fibrotic area to the total tissue area. For each sample, two sections were analyzed, and quantitative analysis was performed using five fields per section at ×40 magnification.

### 2.9 Immunohistochemistry

Immunostaining for Cx40 was performed on formalin‐fixed, paraffin‐embedded atrial tissue sections. Antigen retrieval was carried out using Histofine Antigen Retrieval Solution, pH 9 (Nichirei Biosciences, Tokyo, Japan, 415291) at 95°C for 40 min. Endogenous peroxidase activity was quenched with 3% H_2_O_2_. After blocking with casein blocking solution (Sakura Finetek, Tokyo, Japan, 10‐0040), sections were incubated with rabbit anti‐Cx40 antibody (Abcam, Cambridge, UK, ab313644; 1:2000) for 1 h at room temperature. Subsequently, sections were incubated with Histofine Simple Stain MAX PO (R) (Nichirei Biosciences, 414341) for 30 min. Color development was achieved using Histofine diaminobenzidine solution (Nichirei Biosciences, 725191) for 5 min, followed by counterstaining with Mayer's hematoxylin. Stained slides were examined using a BZ‐X710 and BZ‐X Analyzer software (Keyence). Five samples per group were analyzed, with two sections per sample and five fields per section quantified at ×100 magnification.

### 2.10 Statistical analysis

All statistical analyses were performed using GraphPad Prism version 10 (GraphPad Software, San Diego, CA, USA) and R version 4.3.1 with appropriate packages. For comparisons between two groups, unpaired *t*‐tests with Welch's correction were used for continuous variables. When data were not normally distributed or when sample sizes were small (*n* < 6) and normality could not be reliably assessed, non‐parametric tests were applied; specifically, the Mann–Whitney *U* test was used for two‐group comparisons. The rate of AF inducibility was compared using Fisher's exact test.

Data are presented as mean ± standard deviation (SD). All experiments were independently repeated at least three times. A *p* value <0.05 was considered statistically significant.

## 3 RESULTS

### 3.1 High‐fat diet with L‐NAME induced an HFpEF‐like systemic and cardiac phenotypes

**FIGURE 1:**
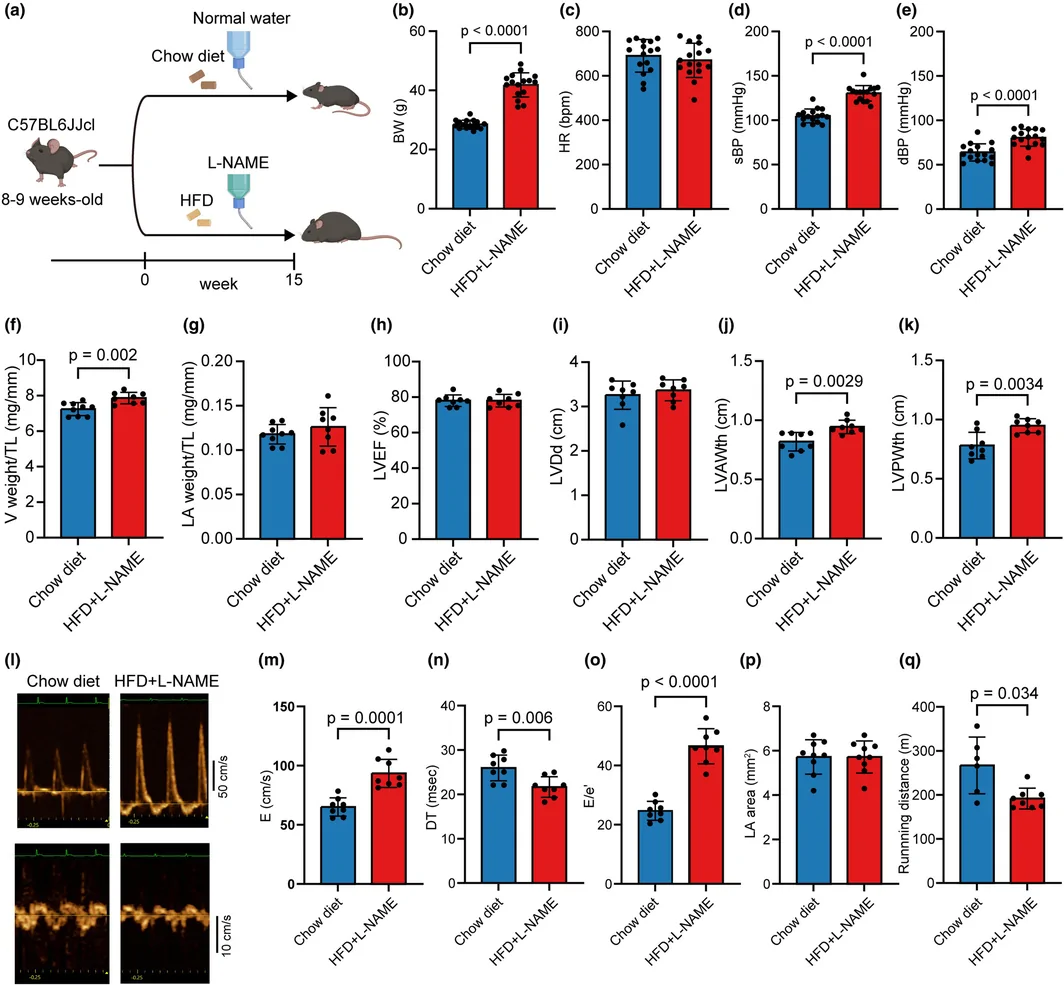
Phenotypic and echocardiographic characterization of HFpEF mice induced by HFD+L‐NAME Mice subjected to a high‐fat diet combined with L‐NAME administration (HFD+L‐NAME) exhibited heart failure with preserved ejection fraction (HFpEF) phenotype characterized by diastolic dysfunction with preserved systolic function. (a) Experimental protocol for induction of the HFpEF model. (b–e) Comparison of body weight (b), heart rate (c), systolic blood pressure (d) and diastolic blood pressure (e) between chow diet‐fed control mice and HFD+L‐NAME mice (Chow diet, *n* = 16; HFD+L‐NAME, *n* = 16). (f, g) Heart weight‐to‐tibial length (HW/TL) ratios in the ventricles (f) and left atrium (g) (Chow diet, *n* = 9; HFD+L‐NAME, *n* = 8). (h–k) Echocardiographic parameters. Left ventricular ejection fraction (LVEF) (h), left ventricular end‐diastolic diameter (LVDd) (i), left ventricular anterior wall thickness (LVAWth) (j) and left ventricular posterior wall thickness (LVPWth) (k) assessed by transthoracic echocardiography (TTE) (Chow diet, *n* = 8; HFD+L‐NAME, *n* = 8). (l) Representative TTE Doppler images. Top panels show transmitral E wave in chow diet–fed control (left) and HFD+L‐NAME mouse (right). Bottom panels show tissue Doppler e' velocity in chow diet–fed control (left) and HFD+L‐NAME mouse (right). (m–o) Quantitative comparison of diastolic function parameters. E‐wave velocity (m), deceleration time (DT) (n), and E/e' ratio (o) assessed by TTE (Chow diet, *n* = 8; HFD+L‐NAME, *n* = 8). (p) Left atrial (LA) area measured by TTE (Chow diet, *n* = 9; HFD+L‐NAME, *n* = 9). (q) Running distance to exhaustion in the treadmill test (TMT) (Chow diet, *n* = 6; HFD+L‐NAME, *n* = 8). Data are presented as mean ± SD. Statistical comparisons were performed using unpaired *t*‐test with Welch's correction.

To validate a previously established metabolic‐ and endothelial dysfunction–based model of HFpEF (Figure 1a), we assessed systemic and cardiac parameters. HFD+L‐NAME mice exhibited a significant increase in body weight, systolic and diastolic blood pressure relative to chow diet‐fed controls, while heart rate remained unchanged (Figure 1b–e), thereby reflecting the combined effects of dietary metabolic overload and nitric oxide synthase inhibition–induced hypertension.

The ventricular heart weight–to–tibial length ratio was significantly elevated in HFD+L‐NAME mice (Figure 1f), indicating the presence of mild concentric ventricular remodeling, whereas the LA weight‐to‐tibial length ratio remained unchanged (Figure 1g). Transthoracic echocardiography showed that left ventricular EF was preserved in both groups (Figure 1h) and that left ventricular end‐diastolic diameter did not differ between groups (Figure 1i), confirming preserved systolic function. HFD+L‐NAME mice exhibited modest increases in left ventricular wall thickness in both anterior and posterior walls (Figure 1j,k), consistent with mild concentric hypertrophy.

In contrast, Doppler echocardiography demonstrated clear evidence of diastolic dysfunction in HFD+L‐NAME mice, as indicated by increased E‐wave velocity, prolonged deceleration time, and an elevated E/e′ ratio (Figure 1l–o). LA area measured by echocardiography did not differ between groups (Figure 1p). In addition, exercise capacity assessed by the treadmill exhaustion test was significantly reduced in HFD+L‐NAME mice, as reflected by shorter running distances to exhaustion (Figure 1q).

Collectively, these data demonstrate that the HFD+L‐NAME protocol reliably induces an HFpEF phenotype characterized by obesity, hypertension, preserved systolic function, diastolic dysfunction, and reduced exercise tolerance.

### 3.2 Increased atrial arrhythmia susceptibility in HFD+L‐NAME mice

**FIGURE 2:**
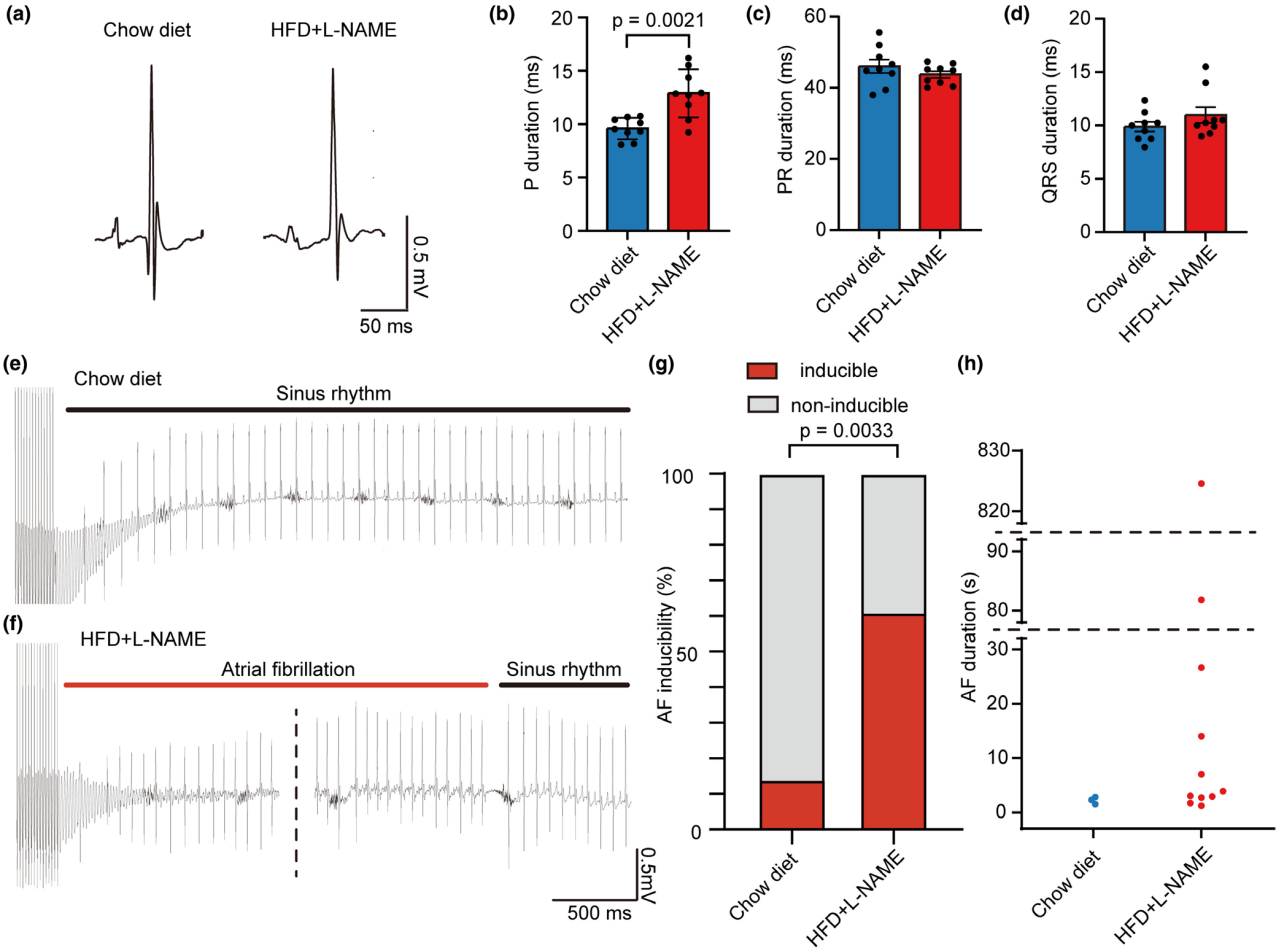
Enhanced atrial arrhythmogenicity and increased AF inducibility in HFpEF mice. HFpEF mice induced by HFD+L‐NAME exhibited atrial electrical remodeling and markedly increased vulnerability to atrial fibrillation (AF) compared with chow diet‐fed controls. (a) Representative surface electrocardiograms from chow diet–fed controls and HFD+L‐NAME mice. (b–d) Quantitative analysis of P wave duration (b), PR interval (c), and QRS duration (d) (Chow diet, *n* = 9; HFD+L‐NAME, *n* = 9). (e, f) Representative surface electrocardiograms during transesophageal atrial pacing showing the absence of AF induction in chow diet mice (e) and successful AF induction in HFD+L‐NAME mice (f). (g, h) Comparison of AF inducibility, defined as the percentage of mice in which AF lasting ≥1 s was induced (g), and AF duration (h) between groups (left: Chow diet, *n* = 21; right: HFD+L‐NAME, *n* = 18). (h) Comparison of AF duration between groups (left: Chow diet, *n* = 3; right: HFD+L‐NAME, *n* = 11). AF induction rates were analyzed using Fisher's exact test, and AF duration was analyzed using the Mann–Whitney U test.

To evaluate atrial electrophysiological remodeling, we first analyzed surface ECG parameters in both groups. Representative ECGs showed distinct morphological differences between chow diet–fed controls and HFD+L‐NAME mice (Figure 2a). Quantitative analysis revealed that P wave duration was significantly prolonged in HFD+L‐NAME mice compared to controls (Figure 2b), indicating impaired atrial conduction. In contrast, PR interval and QRS duration did not differ significantly between the two groups (Figure 2c,d), suggesting that atrioventricular conduction and ventricular depolarization remained largely preserved in this HFpEF model.

To further assess atrial electrophysiological vulnerability, transesophageal atrial pacing was performed to induce atrial arrhythmias. Representative tracings demonstrated the absence of AF induction in chow diet–fed controls (Figure 2e) and successful AF induction in HFD+L‐NAME mice (Figure 2f). AF induction was rare in chow diet–fed controls (3 of 21 mice; 14.3%), whereas the incidence was significantly higher in HFD+L‐NAME mice (11 of 18 mice; 61.1%) (Figure 2g). Moreover, AF duration tended to be longer in the HFD+L‐NAME group than in chow diet–fed controls (Figure 2h).

These results indicate that the HFpEF condition induced by HFD+L‐NAME is associated with atrial electrical remodeling as shown by prolonged P wave duration and marked increase in atrial arrhythmogenic vulnerability.

### 3.3 Optical mapping reveals APD prolongation, slowed conduction, and increased conduction heterogeneity in HFD+L‐NAME atria

**FIGURE 3:**
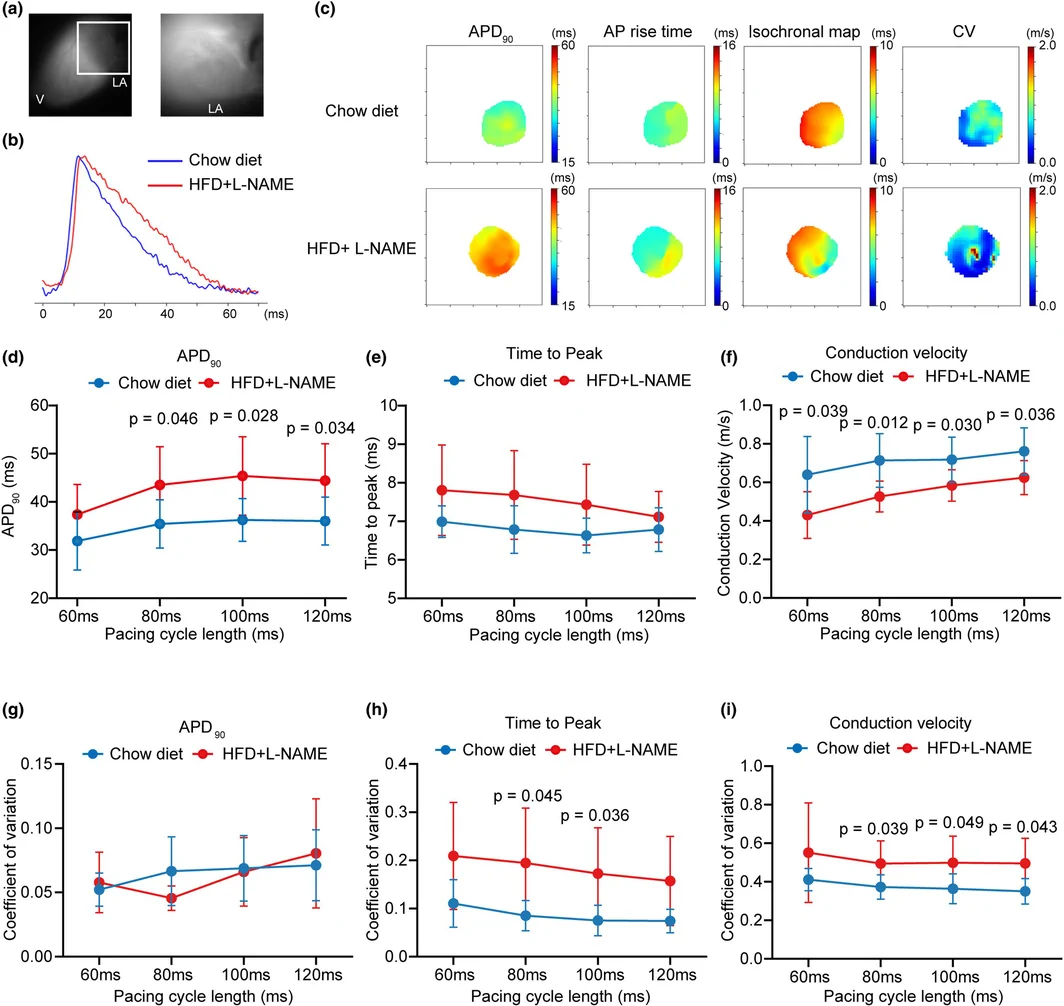
Impaired atrial conduction and increased conduction heterogeneity revealed by optical mapping. Optical mapping demonstrated slowed atrial conduction and increased conduction heterogeneity in HFpEF mice induced by HFD+L‐NAME. (a) Representative raw optical fluorescence images of the heart obtained prior to activation map reconstruction. The left panel shows a wide‐field view including the left atrium and ventricle, and the right panel shows a magnified view of the LA region. (b) Representative action potential (AP) traces recorded from the atrial myocardium in chow diet‐fed mice (blue) and HFD+L‐NAME mice (red). (c) Representative atrial action potential duration (APD) map, AP rise time map, isochronal activation map, and conduction velocity (CV) map from chow diet‐fed control mice (top) and HFD+L‐NAME mice (bottom). (d–f) Quantitative comparisons of APD at 90% repolarization (APD90), AP rise time, and CV at pacing cycle lengths (PCLs) of 60, 80, 100, and 120 ms (blue: Chow diet–fed controls; red: HFD+L‐NAME mice) (*n* = 7 per group). (g–i) Spatial heterogeneity of APD90, AP rise time, and CV expressed as the coefficient of variation (CoV) (blue: Chow diet–fed controls; red: HFD+L‐NAME mice) (*n* = 7 per group). Data are presented as mean ± SD. Statistical comparisons were performed using unpaired *t*‐tests with Welch's correction.

To investigate the electrophysiological substrate underlying enhanced AF vulnerability and to directly characterize atrial electrical remodeling, ex vivo optical mapping was performed in isolated hearts. APD maps and AP rise time maps were constructed from atrial fluorescence signals, while isochronal activation maps were derived from local activation times and subsequently converted into CV maps (Figure 3a–c).

APD was significantly prolonged in HFD+L‐NAME atria compared with controls (Figure 3b), and this prolongation persisted across multiple pacing cycle lengths, indicating global impairment of atrial repolarization (Figure 3d). AP rise time showed a consistent trend toward prolongation in HFD+L‐NAME mice across all pacing rates (Figure 3e), although this did not reach statistical significance. A marked reduction in CV was observed in HFD+L‐NAME atria at all pacing cycle lengths tested, demonstrating generalized slowing of impulse propagation (Figure 3f), consistent with the prolonged P wave duration observed in surfaceECG. Spatial dispersion analysis revealed no significant difference in APD heterogeneity between groups (Figure 3g). AP rise time heterogeneity was modestly increased in the HFD+L‐NAME group at pacing cycle length of 80 ms and 100 ms (Figure 3h). Notably, CV heterogeneity was significantly elevated in HFD+L‐NAME atria at pacing cycle lengths of 80, 100, 120 ms (Figure 3i).

These findings demonstrate that HFpEF mice exhibit a combination of APD prolongation, global conduction slowing, and pronounced conduction heterogeneity, providing a mechanistic electrophysiological substrate capable of supporting the increased AF inducibility observed in vivo.

### 3.4 Selective downregulation of Cx40 in the left atrium without inflammatory or fibrotic activation or transcriptional changes in key ion channel genes

**FIGURE 4:**
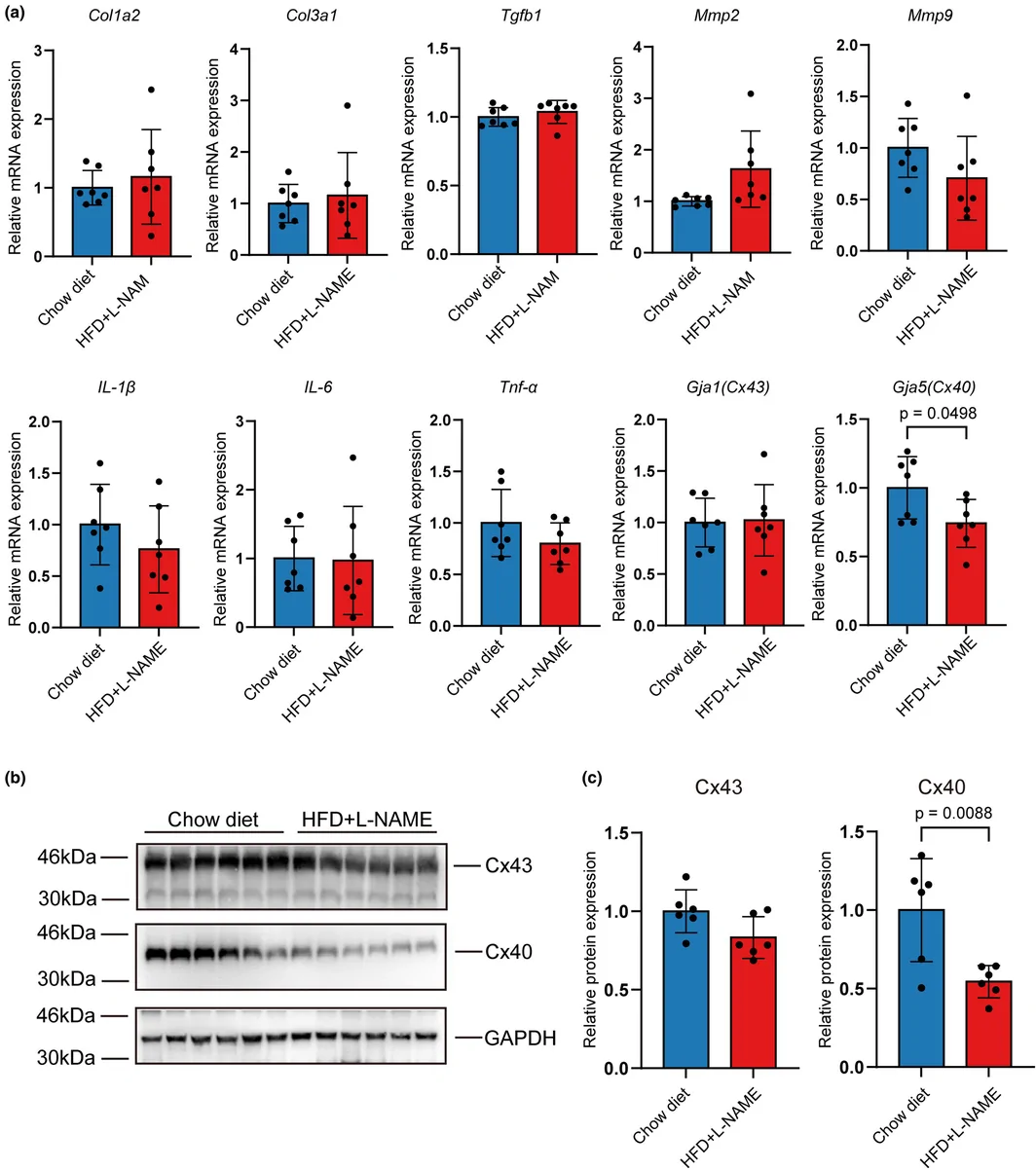
Selective downregulation of connexin 40 expression in the atria of HFpEF mice. Connexin 40 (Cx40) expression was selectively and significantly reduced in the atria of HFpEF mice induced by HFD+L‐NAME. (a) Relative mRNA expression levels of inflammatory markers, fibrotic markers, and gap junction–related genes in atrial tissue assessed by quantitative PCR (*n* = 7 per group). (b, c) Representative western blot images and densitometric quantification of Cx40 and Cx43 protein expression in left atrial tissue from chow diet‐fed control mice and HFD+L‐NAME mice. (*n* = 6 per group). GAPDH was used as a normalized control. Data are presented as mean ± SD. Statistical comparisons were performed using unpaired *t*‐tests with Welch's correction.

To clarify the molecular basis of atrial conduction abnormalities, gene expression analyses were performed in LA tissue. The mRNA levels of inflammatory markers (*Il6*, *Il1b*, and *Tnfa*) and fibrotic markers (*Col1a1*, *Col3a1*, *Tgfb1*, *Mmp2*, and *Mmp9*) did not differ between groups, indicating that neither inflammatory activation nor extracellular matrix remodeling was prominent in this HFpEF model (Figure 4a).

To explore the mechanism underlying APD prolongation, we examined selected genes encoding potassium channel and the L‐type calcium channel in LA tissue. The expression levels of the potassium channel related genes (*Kcnd2*, *Kcna5*, *Kcnh2*, *Kcnj2*, and *Kcnq1*) and L‐type calcium channel gene *Cacna1c* did not significantly differ between chow diet fed and HFD+L‐NAME mice (Figure S1). These findings suggest that APD prolongation was not explained by changes in the selected potassium channel or L‐type calcium channel genes examined at the mRNA level.

In contrast, expression of *Gja5*, encoding *Cx40*, was significantly reduced in HFD+L‐NAME atria, whereas expression of *Gja1* (*Cx43*) remained unchanged (Figure 4a). Consistent with these transcriptional findings, western blot analysis demonstrated a marked reduction in Cx40 protein levels in HFD+L‐NAME atria, while Cx43 protein abundance was preserved (Figure 4b,c and Figure S2).

These results demonstrate that in this HFpEF model, selective downregulation of Cx40 occurs in the absence of inflammatory or fibrotic remodeling, providing a potential mechanism for the observed atrial conduction slowing and increased conduction heterogeneity.

### 3.5 Histology shows preserved atrial fibrosis but reduced atrial Cx40 expression

**FIGURE 5:**
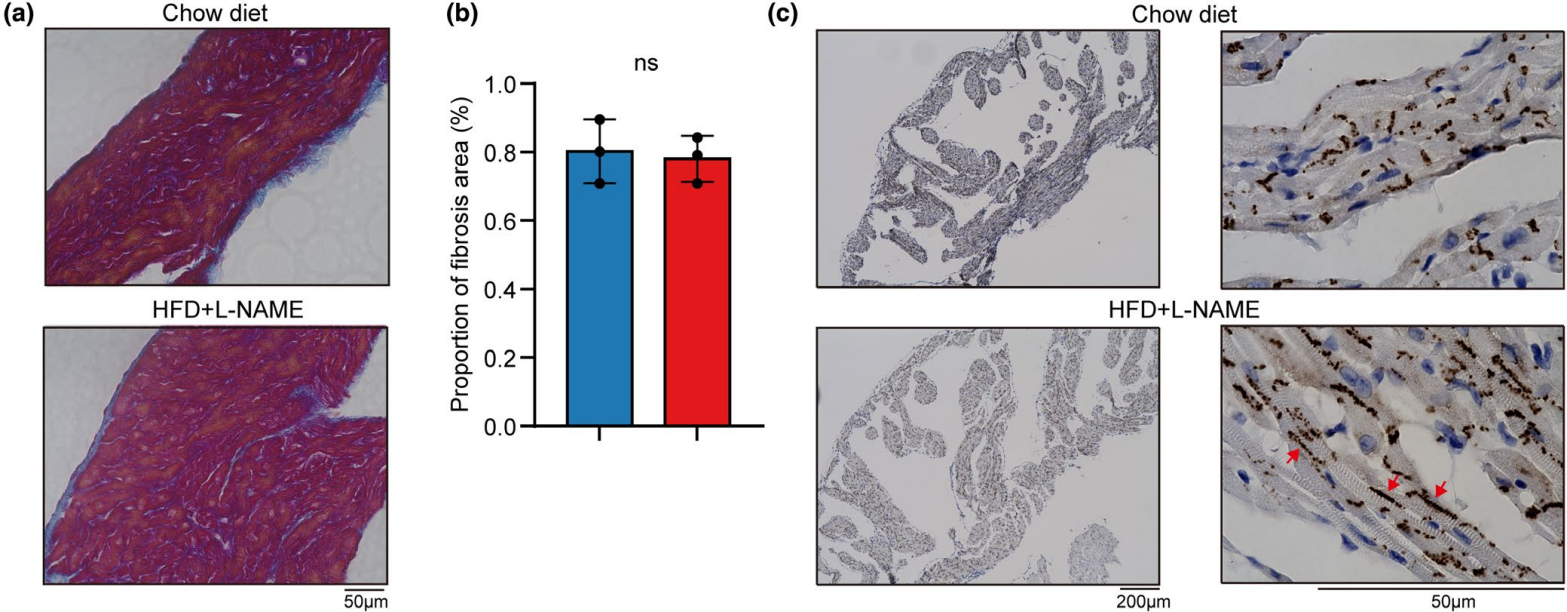
Preserved atrial structure and reduced Cx40 immunoreactivity in HFpEF mice Histological analysis revealed preserved atrial structure with reduced connexin 40 (Cx40) immunoreactivity in HFpEF mice induced by HFD+L‐NAME. (a) Representative Masson's trichrome–stained sections of the left atrium from chow diet‐fed control (40×, top) and HFD+L‐NAME mice (40×, bottom). (b) Quantitative comparison of fibrotic area in the left atrium based on Masson's trichrome staining (*n* = 3 per group). (c) Representative immunohistochemical staining for Cx40 in the left atrium. Low‐magnification images (10×, left panels) and high‐magnification images (100×, right panels) are shown for each group. Red arrows indicate the lateralization of Cx40. Data are presented as mean ± SD. Statistical comparisons were performed using unpaired *t*‐tests with Welch's correction. *p* < 0.05 was considered statistically significant.

Histological evaluation of the left atrium revealed no significant differences in interstitial fibrosis between the chow diet–fed and HFD+L‐NAME groups, as assessed by Masson trichrome staining (Figure 5a,b). Immunohistochemical analysis demonstrated a marked reduction in Cx40 immunoreactivity in the atrial myocardium of HFD+L‐NAME mice compared with chow diet–fed controls, with increased lateralization of Cx40 (Figure 5c, Figure S3). These histological findings are consistent with molecular and functional data and support a role for reduced Cx40 expression in the conduction abnormalities observed in this HFpEF model.

## 4 DISCUSSION

In this study, the main finding is that, in a HFpEF mouse model, atrial conduction abnormalities are observed and atrial arrhythmogenicity is increased. Notably, a selective downregulation of Cx40 may underlie this conduction disturbance, and such electrical remodeling could even precede structural remodeling. Specifically, we observed marked Cx40 downregulation accompanied by decreased conduction velocity and increased conduction heterogeneity in mice exhibiting significantly increased AF inducibility. Importantly, this arrhythmogenic substrate developed in the absence of atrial fibrosis, inflammatory cytokine upregulation, or atrial enlargement.

AF substrate formation is generally considered to involve both electrical and structural remodeling (Nattel et al., 2020), with atrial fibrosis playing a particularly important role in promoting conduction abnormalities (Schotten et al., 2025). Previous studies using various experimental models of heart failure, including myocardial infarction, pressure‐overload, and tachypacing‐induced failure, have demonstrated that atrial inflammation and fibrosis lead to coexistent electrical and structural remodeling (Hanna et al., 2004; Iwamiya et al., 2024; Moe & Armstrong, 1999; Schiattarella et al., 2019). In contrast, our findings in this HFpEF model reveal that substantial electrical remodeling and AF vulnerability can develop before the onset of significant structural changes. This observation may suggest potential pathophysiological differences in AF mechanisms between HFpEF and HFrEF. It should be noted that atrial fibrosis and structural remodeling remain important contributors to AF substrate formation in HFpEF, and it is possible that with longer observation periods, this model may develop progressive fibrosis. Nevertheless, our study provides important evidence that electrical remodeling, particularly Cx40‐mediated gap junction dysfunction, can precede structural changes and contribute to AF vulnerability in HFpEF, potentially offering a critical opportunity for preventive intervention before irreversible structural damage occurs.

In addition to conduction abnormalities, optical mapping revealed APD prolongation in HFD+L‐NAME atria. APD is shaped by the balance between outward potassium currents and inward calcium currents (Varro et al., 2021). Therefore, we examined selected potassium channel and L‐type calcium genes in atrial tissue. No significant differences in the expression of these genes were observed between chow diet‐fed and HFD+L‐NAME mice, suggesting that APD prolongation was not driven by transcriptional changes in the genes examined. APD prolongation may therefore involve functional or post‐transcriptional remodeling of repolarizing currents, including changes in protein abundance, channel phosphorylation, membrane trafficking, or current density (Nattel et al., 2020; Nerbonne & Kass, 2005; Varro et al., 2021). Previous studies using HFpEF models have also implicated enhancement of the late sodium current as a possible contributor to APD prolongation (Hegyi et al., 2022), although this mechanism was not directly assessed in the present study. Beyond its mechanism, APD prolongation may also have important arrhythmogenic consequences. In particular, prolonged APD increases the risk of early afterdepolarizations, which can serve as triggers for ectopic activity and initiate re‐entrant arrhythmias (Iwasaki et al., 2011; Weiss et al., 2010). The HFD+L‐NAME group also showed a trend toward a steeper APD‐rate adaptation curve. Although this trend was not formally quantified, exaggerated rate‐dependent changes in APD may promote repolarization instability and could further interact with conduction slowing and conduction heterogeneity to destabilize the atrial substrate (Qi et al., 2015; Yan et al., 1998). Thus, APD prolongation represents a distinct form of atrial electrical remodeling in this HFpEF model, potentially amplifying arrhythmia susceptibility through both triggered activity and repolarization instability. The mechanisms underlying this remodeling, however, remain to be fully elucidated.

The reduced Cx40 expression observed in this study represents a selective gap junction abnormality, given that Cx43 expression was preserved, suggesting an atrial conduction impairment. This finding is consistent with a previous report in the mouse HFpEF model, which similarly demonstrated downregulation of Cx40 in atrial tissue (Tong et al., 2022). Our data suggest that Cx40 downregulation and mislocalization (Kanagaratnam et al., 2004; Kato et al., 2012; Kostin et al., 2002), both of which have been shown to impair gap junction function, may disrupt atrial conduction in HFpEF, thereby promoting a pro‐arrhythmic substrate even in the absence of structural remodeling. Furthermore, the lack of evidence for aggravated inflammation or significant mechanical stress in this model suggests that Cx40 downregulation may occur through alternative mechanisms. Given that this model is characterized by obesity, hypertension, and nitric oxide synthase inhibition (Schiattarella et al., 2019), potential mechanisms may include oxidative stress and impaired NO signaling, both of which have been implicated in gap junction remodeling in other contexts (Petersen et al., 2017; Yan et al., 2025). Metabolic disturbances associated with obesity, diabetes, and hypertension have been shown to drive atrial conduction remodeling, including connexin downregulation and lateralization (Bode et al., 2024). Therefore, the metabolic milieu of this HFpEF model may similarly contribute to the gap junction remodeling. However, further mechanistic studies are required to definitively establish the molecular pathways responsible for Cx40 downregulation in this HFpEF model.

Several limitations should be acknowledged. First, this study used only male mice to ensure consistent phenotypic expression, as the HFpEF phenotype is markedly attenuated in female mice in this model (Tong et al., 2019); however, sex‐specific mechanisms of atrial remodeling and AF susceptibility in HFpEF warrant future investigation. Second, while our data demonstrate an association between Cx40 downregulation and AF susceptibility, direct causal evidence was not obtained. Further studies using genetic manipulation approaches are needed to confirm causality and to assess the therapeutic potential of Cx40‐targeted strategies. Third, the molecular mechanisms upstream of Cx40 downregulation remain incompletely understood. Fourth, our findings are based on a metabolic HFpEF model and may not fully represent other HFpEF etiologies. Finally, although APD prolongation was observed in HFD+L‐NAME atria, early afterdepolarizations and triggered activity were not directly assessed in the present study, and their contribution to arrhythmogenesis in this model remains to be determined.

In conclusion, in a murine metabolic HFpEF model, we observed significantly increased AF inducibility, marked Cx40 downregulation in the left atrium, and impaired atrial conduction, all in the absence of atrial structural remodeling. Further investigation is warranted to elucidate the mechanisms underlying these electrical alterations and their potential role in HFpEF‐associated AF.

## AUTHOR CONTRIBUTIONS

**Kohei Kawajiri:** Formal analysis; investigation; methodology; software; validation; visualization. **Satoshi Iwamiya:** Formal analysis; investigation; methodology; validation; visualization. **Leo Yaga:** Investigation; validation. **Masahiro Yamazoe:** Investigation; methodology; supervision. **Kensuke Ihara:** Methodology; project administration; supervision; validation. **Yurie Soejima:** Investigation; methodology; resources; supervision; visualization. **Tetsuo Sasano:** Conceptualization; data curation; formal analysis; funding acquisition; investigation; methodology; project administration; resources; supervision.

## FUNDING INFORMATION

This study was partly supported by the Japan Society for the Promotion of Science (JSPS) (No. 22K08152 to T.S.).

## CONFLICT OF INTEREST STATEMENT

The authors declare that they have no conflict of interest.

## ETHICS STATEMENT

All experimental protocols were approved by the Institutional Animal Care and Use Committee of the Institute of Science Tokyo under protocol numbers #A2021‐014 and #A2024‐103.

## Supporting information


Appendix S1.

## Data Availability

The data that support the findings of this study are available from the corresponding author upon reasonable request.
