# Supplementary material for: Selective loss of Cx40 and conduction abnormalities underlie enhanced atrial fibrillation susceptibility in a HFpEF model in male mice
Source: Physiol Rep. 2026 Jun 4;14(11):e70952. doi: 10.14814/phy2.70952 (PMC13581074; doi:10.14814/phy2.70952)
Supplement: Supplementary file 1 [file PHY2-14-e70952-s001.docx]

# Selective loss of Cx40 and conduction abnormalities underlie enhanced atrial fibrillation susceptibility in a HFpEF model in male mice

**Supplemental Appendix**

Supplemental Table 1. Primer sequence used for quantitative PCR…………………………...….2

Supplementary Figure 1. mRNA Expression of potassium channel and calcium-handling genes in atrial tissue. ……………………………………….……………………………….…………..…3

Supplementary Figure 2. Full membrane images of Western blots. ………………….…….……..4

Supplementary Figure 3. Representative images of Cx40 immunostaining in the mouse left atrium. …………………………………………………….……………………………………….……..5

## Supplemental Table 1. Primer sequence used for quantitative PCR.

## Supplemental Figure 1. mRNA Expression of potassium channel and L-type calcium channel genes in atrial tissue.

Relative mRNA expression levels of potassium channel related genes (*Kcnd2, Kcna5, Kcnh2, Kcnj2, and Kcnq1*) and the L-type calcium channel gene *Cacna1c* were measured in atrial tissue by quantitative PCR. No statistically significant differences were observed between the chow diet and high-fat diet (HFD) + L-NAME groups.

Data are presented as mean ± SD. Statistical comparisons were performed using unpaired t-test with Welch’s correction.

## Supplemental Figure 2. Full membrane images of Western blots.

Full-length images of blot for Figure 4b. Cropped area is indicated with red square.

## Supplemental Figure 3. Cx40 immunostaining images of atrial tissue from all samples in each group.

Cx40 immunostaining images from atrial sections of all analyzed mice are shown for the chow diet and high-fat diet (HFD) + L-NAME groups (n = 5 per group). These images provide a visual overview of Cx40 localization patterns across samples. The distribution of Cx40 immunoreactivity was visually consistent with a lateralized localization pattern in atrial tissue.

Scale bars: 20 μm.
